# Intersubject Spatial Pattern Correlations During Movie Viewing Are Stimulus-Driven and Nonuniform Across the Cortex

**DOI:** 10.1093/texcom/tgaa076

**Published:** 2020-10-23

**Authors:** Angela Zhang, Reza Farivar

**Affiliations:** Department of Ophthalmology and Visual Sciences, McGill University, Montreal H3G 1A4, Canada; Department of Ophthalmology and Visual Sciences, McGill University, Montreal H3G 1A4, Canada

**Keywords:** fMRI, intersubject similarity, MVPA, naturalistic stimulation, visual perception

## Abstract

A fundamental step to predicting brain activity in healthy and diseased populations is characterizing the common spatio-temporal response to a shared experience. Multivoxel pattern analysis allows us to investigate information encoding through these patterns; however, we have yet to explore local, stimulus-driven, patterns of cortical activity during naturalistic stimulation. We sought to examine these patterns with minimum interpolation—excluding functional alignment—to characterize the most basic degree of shared response between subjects. We used an unbiased analytic approach, combined with rich, naturalistic, and nonsemantic stimulation to estimate shared spatial patterns in functional magnetic resonance imaging responses across a large group. We found that meso-scale spatial patterns were shared nonuniformly across the visual cortex and represent information distinct from the shared temporal response. Shared spatial patterns were stimulus-driven, modulated by pattern size, and more sensitive to the contrast of 3D versus 2D stimulus differences than the temporal signals. Although the grand functional structure of the brain is understood to be common, these results suggest that even at a meso-scale, we share common spatial structures with anatomical alignment alone. The strength of this similarity varies across the cortex, suggesting some spatial structures are innately organized, whereas others are shaped by factors such as learning and plasticity.

## Introduction

Spatio-temporal patterns across the cortex encode everyday experiences in a common functional architecture. Functional magnetic resonance imaging (fMRI) reveals much of this pattern but classic time-series modeling (I.e., univariate generalized linear model approaches) has been limited in understanding information representation in the cortex (Sereno et al. 1995; DeYoe et al. 1996; Engel et al. 1997). Multivoxel pattern analysis (MVPA) techniques overcome this limit and have been instrumental in assessing how the cortex stores, encodes, and organizes information. Meso-scale responses to features such as color, orientation, and edges can be used to build multivoxel “decoders,” which can even serve to estimate the experience of the subject (Naselaris et al. 2009; Zurawel et al. 2016). Spatial patterns can carry category (Haxby et al. 2001) or task-related information (Kriegeskorte et al. 2006), they can be robust even in the absence of conscious perception (Sterzer et al. 2008; Schurger et al. 2010), and these findings can generalize to nonvisual modalities (Abrams et al. 2011). Some have suggested adapting MVPA to the study of intersubject similarity, so we could also measure common encoding mechanisms and representations in the population (Cohen et al. 2017; Nastase et al. 2019), similar to past studies using the temporal responses (Hasson et al. 2004). We sought to accomplish this by characterizing intersubject similarity using local spatial patterns of the cortex during naturalistic stimuli. Response patterns shared across individuals would implicate common underlying brain structures and, once characterized, would provide the basis of predicting brain activity in both healthy and diseased populations.

The combination of intersubject similarity measures with naturalistic viewing paradigms, such as the method of intersubject correlation (“ISC”; Hasson et al. 2004), first demonstrated that the shared temporal structure across subjects can be measured even during such naturalistic conditions as freely watching a movie. Naturalistic stimuli can be advantageous because while artificial and highly controlled stimuli are designed for group comparisons, their results may not be ecologically valid. There is growing evidence that natural and artificial stimuli result in different responses in the cortex (Basole et al. 2003; Felsen et al. 2005; Koene and Zhaoping 2007). Task-based paradigms may also impose top-down influence that drives generalizable spatial patterns only during the specific conditions of the task (Anllo-Vento et al. 1998; Williams et al. 2007). Even naturalistic stimuli can have top-down influences through narrative structure that drive common spatial patterns, potentially obstructing, or confounding stimulus-driven patterns (Englander et al. 2012; Naci et al. 2014; Lu et al. 2016). To characterize stimulus-driven shared cortical patterns, we used a task-free naturalistic stimulus that does not include narrative structure and includes central fixation to minimize top-down attentional and emotional influences. This simplification, compared to prior intersubject similarity studies on the temporal structure, allows us to probe the most basic degree of shared local spatial structure across subjects before later defining its more complex parameters.

Until recently, naturalistic stimuli could not be used with spatial approaches such as MVPA to assess commonality due to the limitations of subject-specific classifiers and poor between-subject generalization. Subject-specific classifiers blind us to shared spatial patterns, potentially losing information about the shared architecture that may be useful to determine fundamental cortical elements. Even with search-light methods to derive local classification performance metrics and then summarizing these maps by alignment across subjects, we would obtain only a map of local performance, not local patterns of activity, which may relate more directly to stimulus-driven representations. In contrast, transposing classifiers from 1 subject to another has been claimed to be unreliable (Cox and Savoy 2003; Haxby et al. 2011), due to the loose relationship between functional and structural topography of the cortex (Watson et al. 1993; Tootell et al. 1995; Tahmasebi et al. 2012; Langs et al. 2016). When between-subject multivoxel pattern classification (bsMVPC) is attempted on the cortical response itself, almost no significant accuracy is found (see Guntupalli et al. 2016, Fig. 2*a*). There are now 2 prominent methods to overcoming these barriers to characterizing common spatial patterns between subjects. The first is using second-order statistics, and the second is functional alignment techniques. We will explain the advantages and disadvantages of each of these methods below, and how the present study fits within this literature.

Between-subject comparisons with MVPA can be enhanced by using representational similarity analysis (RSA) to abstract the response pattern to a “signature” of the cortical representation (Kriegeskorte et al. 2008; see Guntupalli et al. 2016), Figure 3*a*, or using large-scale patterns covering much of the cortex (Shinkareva et al. 2008; Shinkareva et al. 2011). Large-scale patterns do not capture information representations of the cortex, but rather, represent the global activity of functional modules. In contrast, RSA reveals distinct information in spatial representations across subjects, even during naturalistic stimulation (Chen et al. 2020). RSA and other second-order techniques (e.g., shared-response model) are extremely useful when comparing and integrating between methods, conditions, models, and populations. However, this technique does not allow for the direct characterization of the stimulus-driven cortical response. The current study directly uses the BOLD response to characterize local spatial pattern similarity across subjects and, in doing so, reveals that shared cortical responses to naturalistic stimuli are nonuniform across the cortex.

Another way to enhance between-subject comparisons with MVPA is to enforce better functional correspondence between subjects. Several methods have been developed to functionally align subjects either by their time series alone, or the full spatio-temporal response together, called “hyperalignment” (Sabuncu et al. 2010; Haxby et al. 2011; Guntupalli et al. 2016). Other methods include modeling a shared response across subjects using functional data, or incorporating functional connectivity to surface-based alignment, among others (Robinson et al. 2014; Chen et al. 2015; Nenning et al. 2017).

With the increasing number of choices for functional alignment comes an increased uncertainty of the effects of each method on the cortical response. For example, if the functional alignment method uses searchlights over the whole brain, such as with hyperalignment, then the size of the searchlight used will influence the alignment outcome. Moreover, this localized approach ignores more large-scale patterns in functional correspondence, such as the effect of cognitive processing level (Tahmasebi et al. 2012). Meanwhile, the mere act of re-alignment necessitates interpolation, thereby disrupting the local measured responses of the cortex and shifting focus onto distributed population responses, which may not be ideal in all cases. We measured local cortical responses with anatomical alignment alone to demonstrate that shared meso-scale representations can indeed be found before functional alignment and thus can be included in future functional alignment techniques to preserve as much of the stimulus-driven response as possible before applying transformations to said responses.

In sum, using MVPA methods on naturalistic stimuli to characterize and potentially predict shared cortical spatial patterns can be best achieved by first establishing the degree and distribution of common patterns with minimal alterations from the stimulus-driven activity. We designed our study to have the least amount of interpolation by directly analyzing the cortical response patterns (first-order) and without functional alignment while still using naturalistic stimuli. We measured a large number of subjects, while they watched non-narrative movie scenes that minimized top-down influences, then analyzed with an unbiased method to determine the distribution of shared cortical spatial patterns. We were able to verify that our estimate of shared cortical patterns was indeed stimulus-driven and not simply an epiphenomenon of anatomical alignment. Importantly, we find that the distribution is not uniform, and these results have important implications for future MVPA studies.

## Materials and Methods

### Procedure and Stimuli

Fifty-four subjects were recruited from the McGill student community (27 females, 27 males, ages 18–44, mean age 25.3) and provided informed written consent in accordance with the Code of Ethics of the World Medical Association (Declaration of Helsinki) and approved by the Research Ethics Board of the McGill University Health Center. All participants were right-handed and had normal or corrected-to-normal vision and were able to perceive depth in stereoscopic 3D movies. Participants watched 2 clips compiled from “Under the Sea 3-D: IMAX” (Hall 2009) during an fMRI scan. Each clip was of 5 min in length and was presented in monoscopic (2D) and stereoscopic (3D), totaling 4 clips and 20 min of viewing time. Clips were presented in random order to each subject. The 3D clips were presented on an magnetic resonance-compatible liquid crystal display screen (1920 × 1080 resolution; BOLDscreen 3D by Cambridge Research Systems Ltd, United Kingdom) and viewed through circular polarizers. The video subtended 17° × 9.4°. The display of the stimulus and synchronization of the stimulus to acquisition start time was done using Stereoscopic Player (http://www.3dtv.at) controlled with MATLAB r2014b (The MathWorks, Inc.) via ActiveX. To isolate visual processes in this study, no audio was included with the stimulus. A white fixation cross was presented at the center of the screen for the entire duration of the stimuli and participants were instructed to fixate on the cross. Although free viewing is preferable for many naturalistic stimuli studies for its improved engagement and similarity to real-world vision, it is not compatible with our analysis. Fixation is necessary to enforce comparability between subjects in our study because we are looking at local spatial patterns of activation on the cortex, meaning the stimulus presented to those receptive fields must match as closely as possible between subjects in order to compare the pattern of response. Each clip was preceded with 4 s of a blank black screen including the fixation point. There was also a small flickering pattern at the bottom right corner of the screen that was used in another experiment for timing purposes. The entire scan lasted approximately 45 min, after which we asked each subject whether they saw two of the clips in 3D.

### Data Acquisition

Acquisition was performed on a full-body 3T Siemens TIM Trio with a 32-channel head coil for anatomical images and 20-channel posterior coil for functional images. Functional images were acquired using a T2*-weighted BOLD sequence (resolution = 3 mm^3^, repetition time (TR) = 2000 ms, echo time (TE) = 30 ms, flip angle = 76, matrix size = 64 × 64, field of view (FOV) = 192 × 192, number of slices = 37, slice thickness = 3 mm), and anatomical images were acquired using a T1-weighted multi-echo magnetization prepared - rapid gradient echo (MEMPRAGE) sequence (resolution = 1 mm^3^). Preprocessing of fMRI data was performed using analysis of functional neuroImages (AFNI) (Cox 1996), with special attention paid to minimize spatial blurring. Specifically, after slice-time correction, all spatial transformations (i.e., motion correction, distortion correction, and registration to anatomical image) were concatenated and applied in a single step. The time series was additionally detrended and denoised by the ANATICOR model (Jo et al. 2010). This denoising model computes nuisance regressors in neighborhoods of white matter voxels to remove local structured noise sources from the fMRI time series. Surfaces were extracted using Freesurfer (http://surfer.nmr.mgh.harvard.edu/) and visually inspected and subsequently manually corrected for errors in the calcarine cortex. We chose to conduct surface-based group level analyses, which offer greater statistical power due to better domain-matching across subjects while preserving individual subjects’ topology (Saad and Reynolds 2012). The resulting surface meshes (36 000 nodes on each hemisphere) of each subject can then be directly compared, whereas the topology of the original surface is preserved and each node on 1 subject’s mesh corresponds to the same node on another subject.

### Intersubject Spatial Pattern Correlation

We calculated the ISC of the spatial patterns inside searchlights that were centered on each surface node of the brain, similar to previous studies (Chen et al. 2016b; Zadbood et al. 2017; Nastase et al. 2019). Searchlights were generated using AFNI’s ROIgrow function at 5 different searchlight radii 3, 5, 7, 9, and 11 mm, but the 3 mm radius searchlights at times contained too few nodes for statistical analysis (e.g., only 4 nodes) and hence was dropped from further analysis. In attempting to constrain our analyses to local and relatively fine-scale spatial patterns, we favored the smaller searchlight sizes and thus also excluded the 11 mm searchlight from further analysis.

**Figure 1:**
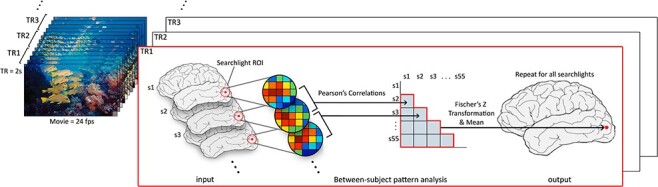
At each TR (leftmost panels of movie frames) of the scan during movie viewing, we calculated the spatial pattern correlation (Pearson’s *r*) between subjects within the same searchlight (red circle on brain) and calculated the group mean (lower triangle of ISC matrix). This value is then projected back onto a template brain (rightmost brain) at the central node of the searchlight (red dot), and the process is repeated for all searchlights and for all TRs. Lastly, the correlations are aggregated over time by averaging over TRs, producing the final estimate of ISPC.

For each node, we calculated Pearson’s correlation between every pair of subjects at every time point and stored the z values—all statistical inferences used these Z-transformed values. The mean of the upper triangle of this square matrix at a given time point then represents the intersubject spatial pattern correlation (ISPC) at that node (see Fig. 1). This group level mean of pairwise correlations can also be interpreted as a measure of the similarity of any individual subject to the mean of all other subjects, or alternatively, how well an individual can be aligned by their local spatial pattern to all other subjects.

A nonparametric significance test is suitable for naturalistic stimuli because it requires minimal assumptions about the data, deals with the multiple comparisons problem over the whole brain and does not require an a priori experimental model. The spatial pattern inside each searchlight was permutated 10 000 times using a Fourier phase randomization routine adapted from Prichard and Theiler (1994), which randomizes the phase of the components while retaining the global power. This method is frequently used in visual object recognition studies as a control condition because it scrambles an image while retaining many of the original visual properties, including higher-order statistics (Nichols and Holmes 2001; Nichols and Hayasaka 2003). It has also been used on magnetoencephalography time series to create a null distribution while respecting time-series statistics such as autocorrelation (Chang et al. 2015). We chose to adapt the Fourier-scrambling method for our spatial pattern analysis because it embodies our null hypothesis the most accurately—that there is no common spatial pattern found between subjects in each searchlight, therefore scrambling the pattern inside the searchlight will have no effect on ISPC. The null hypothesis treats subjects as a random effect because there is no relationship between subjects, and this method of scrambling disrupts only local activation patterns. For each permutation, the Pearson’s correlation coefficient was measured in the same manner as in the real data. Each permutation iteration involved taking the maximum correlation coefficient across all searchlights over the entire brain. The null distribution is therefore made up of the 10 000 maximums, one for each permutation iteration. Since the null distribution is composed of absolute maximums, we strictly control for family-wise error rate (Nichols and Holmes 2001; Nichols and Hayasaka 2003). Each of the 4 movie clips received its own set of 10 000 permutations. A single threshold test for significance was performed using the 95th percentile of each null distribution, as outlined in Nichols and Holmes (2001), and applied to ISPC results of each movie clip. More specifically, each movie clip produced 1 set of ISPC coefficients, 1 value for each node on the cortical surface, and coefficients, which fell above the significance threshold were deemed significant. Alternate methods of conducting ISCs have been outlined in Chen et al. (2016a), who suggest subject-wise bootstrapping over element-wise permutations; however, past literature on intersubject temporal correlations during movie viewing tend to use scene scrambling (a type of element-wise permutation method), so we used this method as well for greater comparability.

### Data Visualization

All significant searchlights were projected onto the Standard-60 mesh (in AFNI’s SUMA) by assigning the mean spatial pattern correlation across time of the searchlight to the central surface node of the searchlight. We used the Python toolbox “Pycortex” (Gao et al. 2015) for final surface visualizations. Visual areas were defined by the Wang et al. (2015) probabilistic atlas composed of 25 topographic maps. For all subsequent analysis, we report data generated from 9 mm searchlights.

### ISPC Variation May Relate to Other Known Maps of the Visual System

To compare spatial pattern similarity across visual areas, we used a sub-sampling method on the pairwise correlation coefficients to generate 27 subsamples of our 53 subject pairs. The subsampling was performed on the upper triangle of the pairwise correlation matrix composed of z-scores, calculated as a part of the ISPC analysis described previously. For each surface node and each time point, subsamples were obtained by randomly sampling the pairs without replacement. Once all subsample groups were created, the z-scores were averaged across pairs within a subsample, and finally, averaged over time points. The resulting values are analogous to ISPC, except, instead of 1 group-level value describing intersubject similarity per node, we have a value for each of our 27 subsamples. Subsampling allowed us to estimate a measure of reliability of our intersubject pattern correlation—regions that have high intersubject pattern correlation but low reliability would exhibit higher variability in the correlation estimate across subsamples. We compared ISPC values between 21 visual areas defined by the probabilistic atlas of Wang et al. (2015) (we concatenated the dorsal and ventral portions of V1, V2, and V3 individually) using pairwise *t*-tests with Benjamini–Hochberg false discovery rate (FDR) (BH-FDR) correction (Benjamini and Hochberg 1995). Due to overlap in searchlights at the borders of visual areas, our comparisons between these areas may be reducing the effect size of differences between visual areas; however, due to our hypothesis that visual areas will exhibit significant differences in ISPC, we chose not to remove these searchlights and instead include as much data as possible. Of particular interest was the comparison between V1, V2, and V3 due to their similarly robust retinotopic organization known to be similar across individuals, and thus a comparison between them would resolve whether variation in ISPC was related to retinotopic mapping.

The visual pathways may also explain variation in ISPC. We compared early (V1, V2, and V3), ventral stream (V4, LO1, LO2, VO1, VO2, PHC1, and PHC2), and dorsal stream (V3a, V3b, hMT, MST, IPS0-IPS5, and SPL1) areas using 2-sample *t*-tests with BH-FDR correction on our 27 subsamples.

### ISPC Variation Is Not a Consequence of Anatomical Alignment

To account for the possibility that the variation in spatial pattern similarity in different visual areas was due to differences in the anatomical alignment between subjects, we compared anatomical alignment with ISPC. Since all group analyses were done on SUMA-generated surfaces, individual surface geometry was conserved while maintaining node correspondence. At each node, the surface convexity describes the height and depth of sulci and gyri. We took the convexity at each node and calculated the variance across subjects. High variance would indicate that the specified node is on different topological locations for different subjects. We smoothed the results with the same searchlight size as used in the ISPC analysis, then calculated the Pearson’s correlation coefficient between convexity variance and ISPC across the visual cortex to determine whether topological variance could predict spatial pattern similarity. This control measure is different from past literature describing the decoupling of functional and anatomical structure (Watson et al. 1993; Tootell et al. 1995; Tahmasebi et al. 2012) because it assumes that even with poor functional alignment during anatomical alignment alone, small-scale functional spatial patterns may still be shared across subjects.

### ISPC Is Not Driven by High Amplitude BOLD Signals

Since ISPC is calculated at each time point, we tested whether time points with high ISPC correlate with time points of high BOLD signal amplitude in order to determine whether spatial pattern similarity is driven by global clusters of high amplitude BOLD signal. An example is if voxels from a small patch of cortex all exhibit uniformly high BOLD signal, it may artificially drive high ISPC and thus would not represent any additional information from temporal pattern similarity. We calculated the FDR corrected *P* value and correlation coefficient between mean BOLD amplitude across subjects over a searchlight to ISPC in the same searchlight.

### Effect of Stereoscopic (3D) Viewing on ISPC

The effect of 3D viewing on spatial pattern correlation was calculated within each visual area. The ISPC in the two 2D clips were first averaged, likewise with the 3D clips. The difference in spatial pattern similarity between the 2D and 3D clips was converted into a z-score and significance was tested with 10 000 permutations involving scrambling of the 2D and 3D labels. The *P* values were BH-FDR corrected.

### ISPC Is Stimulus-Dependent

Lastly, to determine the stimulus-dependent nature of the spatial patterns, we calculated the similarity in spatial patterns between 2 clips (the same clip but presented in 2D and 3D, or different clips altogether presented in only 3D). For each clip, we averaged the spatial pattern across subjects, then calculated the Pearson’s correlation coefficient between clips and averaged the values over time. To determine a significance threshold, we computed 1000 permutations of the time-series within each searchlight such that the spatial pattern in a specific time point was not altered, but the position of that pattern in the time series was scrambled. We used the 95th percentile of the permutation distribution, FDR-corrected the *P* values, and thresholded the data at *P* = 0.05. The same analysis was conducted on the BOLD time series, smoothed with the same searchlight size as in the spatial pattern version, and averaged over subjects before calculating the correlation coefficient. Permutations of the BOLD temporal response included phase randomization of the time series at each node as to not disrupt temporal autocorrelation, followed by multiple comparisons correction in the same manner as the spatial response.

## Results

### Spatial Patterns Are Shared across Individuals and Vary across the Cortex

**Figure 2:**
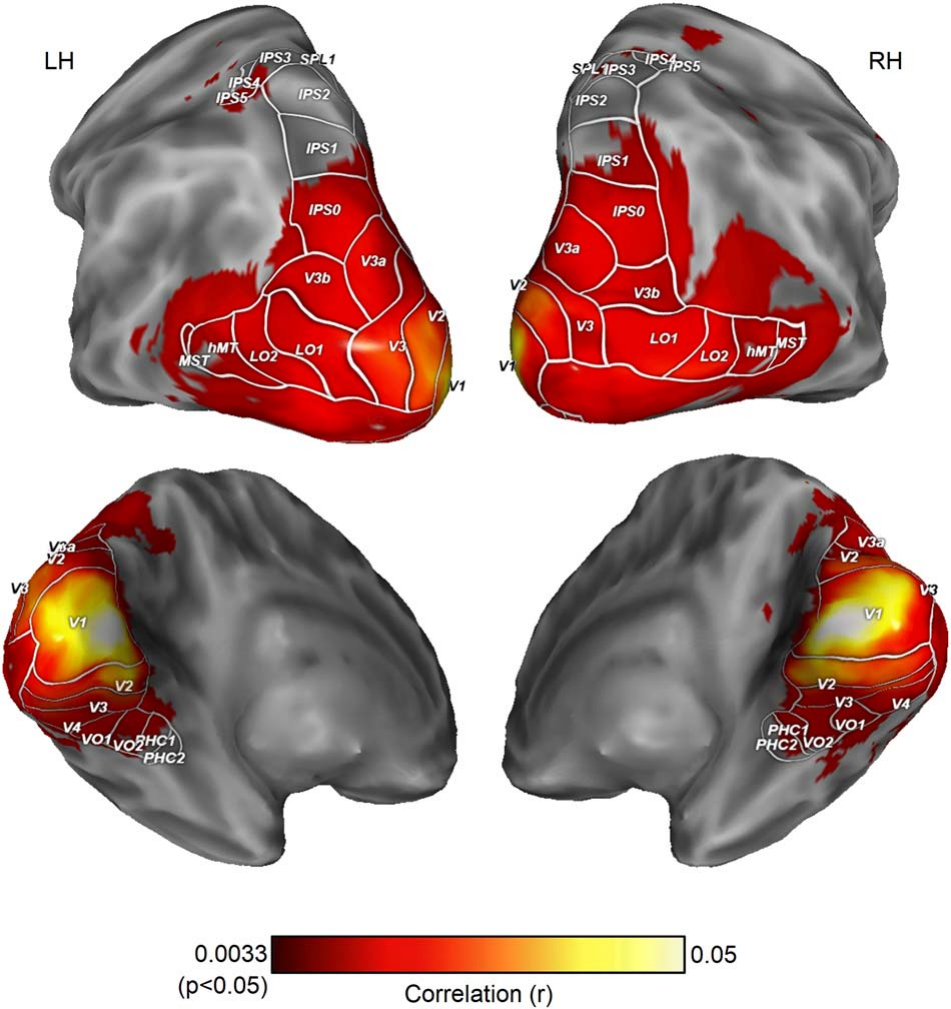
ISPC across subjects in 2D version of clip 1, analyzed with 9 mm searchlights (N = 54). Significant similarity was found across most of the visual cortex except late dorsal and ventral areas and is not uniform across the cortex. Only areas that passed the family-wise error corrected threshold from permutation tests are shown. Visual area borders reflect population atlas boundaries (Wang et al. 2015).

Spatial patterns are similar between subjects, robust, and vary in similarity as a function of cortical hierarchy (Fig. 2). The conservative permutation test with family-wise error correction revealed that up to 10% of the cortex had significant ISPC across subjects. For 5 mm radius searchlights, 2.7% of the cortex exhibited significant ISPC. For 7 mm, 7.7% and for 9 mm it was 10.0%. These results suggest that shared spatial patterns have a range of sizes, with a minimum 7–9 mm radius in most visual areas and as small as 5 mm in early visual cortex (see Supplementary Figs 1 and 2 for data on 5 mm and 7 mm searchlights).

**Figure 3:**
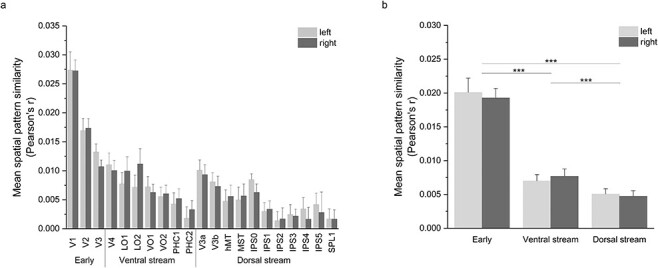
Mean ISPC of subsampled groups in each (*a*) visual area and (*b*) visual stream for clip 1 in 2D with searchlight of 9 mm using N = 27 sub-samples of 53 pairs each. Spatial patterns similarity was variable across visual areas (*a*). Two-sample *t*-tests showed significant differences between many of the visual areas (see Supplementary Fig. 3 for pairwise comparisons of areas) and between all visual streams (*b*). Error bars represent standard deviation. ^***^Represents *P* < 0.001.

Significant ISPC was found in all visual areas except in late dorsal and ventral visual areas, namely intraparietal sulcus (IPS1—IPS4), superior parietal lobule 1 (SPL1) and posterior parahippocampal cortex (PHC1, PHC2)—even larger searchlights (i.e., 7 mm and 9 mm radii) failed to reveal shared patterns across subjects in these areas (Fig. 3*a*).

Spatial pattern similarity is not uniform across the visual cortex. We first tested whether segregation by visual pathways explains the variation. Using the probabilistic atlas of Wang et al. (2015), we first compared the average ISPC in areas designated as early (V1, V2, and V3), ventral stream (V4, LO1, LO2, VO1, VO2, PHC1, and PHC2), or dorsal stream (V3a, V3b, hMT, MST, IPS0-IPS5, and SPL1) with 2-sample *t*-tests. For both left hemisphere (LH) and right hemisphere (RH), early visual areas [LH M = 0.02, standard deviation {SD} = 0.002; RH M = 0.02, SD = 0.001] have significantly higher correlation than dorsal stream (LH M = 0.005, SD = 0.0008; RH M = 0.005, SD = 0.0008; *t*(52) = 34.67, *P* < 1 × 10^−10^ and *t*(52) = 47.06, *P* < 1 × 10^−10^, respectively, for LH and RH) and ventral stream (LH M = 0.007, SD = 0.0009; RH M = 0.008, SD = 0.0008; *t*(52) = 29.40, *P* < 1 × 10^−10^, and *t*(52) = 34.26, *P* < 1 × 10^−10^, respectively, for LH and RH, Fig. 3*b*).

Between Dorsal and Ventral areas, there was also a significant difference, with Dorsal areas exhibiting the lowest pattern similarity (*t*(52) = 8.34, *P* < 1 × 10^−10^ and *t*(52) = 11.33, *P* < 1 × 10^−10^, respectively, for LH and RH, Fig. 3*b*).

We then asked if the pattern of ISPC across the cortex is related to retinotopy—a putatively intrinsic organizing principle that is known to be similar across individuals. To answer this question we compared ISPC across the 3 areas with robust retinotopic organization—V1, V2, and V3. We found significant differences in the magnitude of similarity between these areas (*t*(52) = 14.27, *P* < 1 × 10^−10^ between V1 and V2, *t*(52) = 21.25, *P* < 1 × 10^−10^ between V1 and V3, and *t*(52) = 7.56, *P* < 1 × 10^−9^ between V2 and V3), suggesting spatial pattern similarity is not directly related to previously-known shared spatial structures such as retinotopic maps (see Supplementary Fig. 3 for all visual area comparisons).

### Spatial Pattern Correlation Is Not Due to Anatomical Misalignment nor BOLD Amplitude

We tested whether spatial pattern similarity is dictated by anatomical alignment—it could simply be the case that when subject anatomies are slightly misaligned, their functional structures are similarly misaligned. To test this, we carried out a correlation analysis between intersubject variability in anatomical convexity determined from the reconstructed surfaces and the ISPC at each node. There was no significant correlation between anatomical alignment and magnitude of ISPC across the brain (Pearson’s *r* = 8.8 × 10^−4^, *P* = 0.96), suggesting our new metric is not explained by anatomical alignment or misalignment.

**Figure 4:**
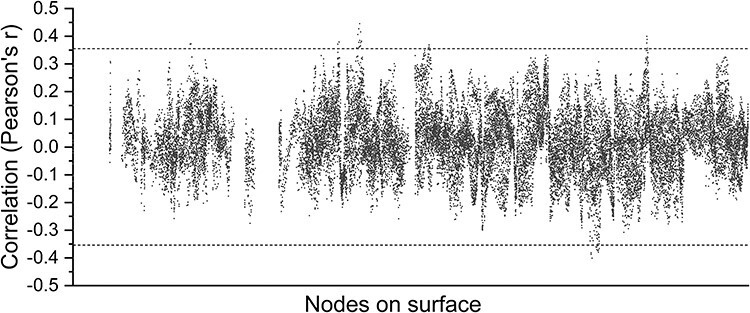
Relationship between mean BOLD signal amplitude and spatial pattern similarity in the RH (N = 54). Dotted line represents threshold at FDR-corrected *P* = 0.05. Almost all nodes are not significantly correlated, indicating weak or no relationship between spatial pattern similarity and BOLD signal amplitude over time.

Our approach to estimating spatial patterns allowed us to obtain a metric per time point, so we wondered whether the time course of ISPC was related to the BOLD time series. We tested the correlation between the 2 measures at every node in the cortex and found that the two are “not” related to each other (Fig. 4). This means that spatial pattern similarity is also not likely explained by BOLD temporal signal-to-noise ratio (SNR)—high BOLD would predict higher SNR, and if ISPC was driven largely by the presence or absence of sufficient BOLD SNR, we would expect the two to be correlated, but that was not observed.

### Spatial Pattern Similarity Is Stimulus-Dependent

**Figure 5:**
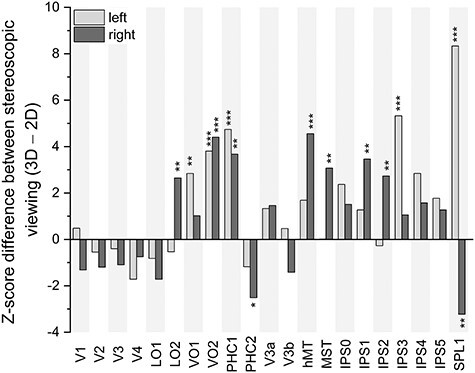
Effect of stereoscopic (3D) versus monoscopic (2D) viewing on ISPC in each visual area. Clip 1 and clip 2 were concatenated for each viewing condition. Significantly greater spatial pattern similarity was observed in higher order areas during stereoscopic (3D) viewing. *Represents *P* < 0.05, **represents *P* < 0.01, and ***represents *P* < 0.001 (N = 54).

Because our participants had viewed the clips in both 2D and 3D conditions, we could use the evoked patterns across different viewings to determine the extent to which stimulus features affected spatial pattern similarity. Viewing of the movie clip in 3D tended to increase ISPC magnitude in several higher level dorsal and ventral areas (Fig. 5). Surprisingly, we found that right PHC2 and right SPL1 exhibit significantly higher ISPC in 2D viewing.

**Figure 6:**
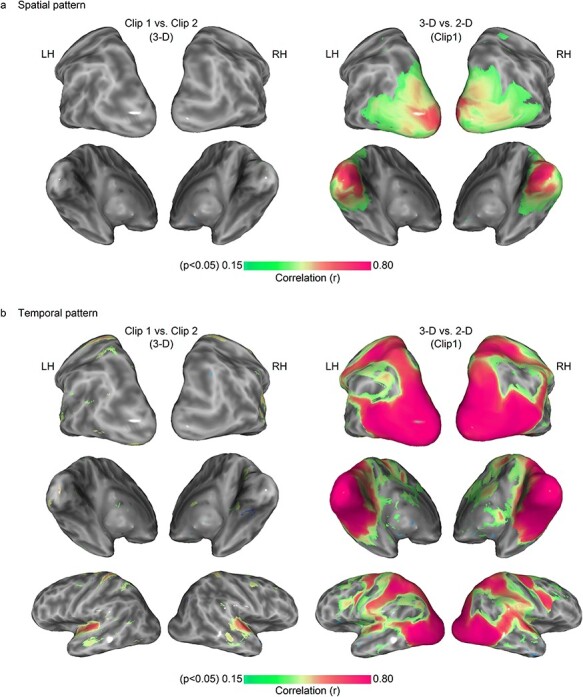
Test of stimulus-dependence of (*a*) spatial pattern and (*b*) temporal pattern consistency. The group-averaged spatial pattern (*a*) was not similar between clip 1 and clip 2 across the whole cortex (left column), but this was not the case for the contrast of 3D versus 2D of the same clip (right column), suggesting spatial patterns are sensitive to even subtle stimulus changes such as the addition of disparity. The analysis was repeated for the temporal pattern (*b*), where similarity between clip 1 and clip 2 in the auditory cortex was observed. Temporal patterns were more comparable across the cortex than spatial patterns in the 3D versus 2D condition, suggesting that spatial patterns may be more sensitive to small stimulus differences. N = 54.

Spatial patterns generated by the same movie clip were very similar, whereas different movie clips generate different spatial patterns (Fig. 6*a*). This result demonstrates that the spatial patterns sampled to generate the ISPC map are clearly stimulus-driven and not a consequence of spontaneous patterns in the brain. In clip 1 versus clip 2 condition, 0.03% of nodes survived significance testing, whereas 9.9% of nodes survived in the 3D versus 2D condition. This further lends support to our hypothesis that spatial patterns represent information encoding in the cortex and so common spatial patterns across subjects represent common functional structures. When looking at the analogous analysis with BOLD temporal ISC, the difference between same and different movie clips is also very apparent (5.5% of nodes are significant in the clip 1 versus clip 2 condition and 51% of nodes are significant in the 3D versus 2D condition; Fig. 6*b*). Interestingly, the magnitude of similarity in the 3D versus 2D condition is much higher in the temporal version than in the spatial version. This suggests that spatial patterns are more sensitive to stereoscopic differences than BOLD temporal patterns–there are greater differences between spatial patterns than between temporal patterns when the only difference between clips is stereoscopy. We also found correlations between the temporal pattern of clip 1 and clip 2 in the superior temporal cortex.

## Discussion

We examined intersubject spatial pattern similarity across the visual system during naturalistic stimulation to determine whether shared patterns were significantly present with anatomical alignment alone and to examine their characteristics. We demonstrate that ISPC is robust, varies across the cortex as a function of visual hierarchy, and is modulated by the stimulus content and stereoscopy even to a greater extent than temporal ISCs. In addition, we verified that ISPC was not explained by variations in anatomical alignment across the cortex or changes in BOLD amplitude over time.

Our study utilized rich naturalistic stimulation under different viewing conditions and in a large cohort of subjects, analyzed using rigorous statistical criteria. We used greater than the minimum number of subjects required for reliability (N > 30, according to Pajula and Tohka 2016)) and strict significance testing with minimal assumptions (Nichols and Hayasaka 2003). We minimized top-down modulations by using a visually rich stimulus that was devoid of narrative structure and enforced comparability with central fixation (Naci et al. 2014; Lu et al. 2016). We also included 3D viewing of the stimulus to mimic real-world vision as closely as possible and compared the responses to 2D viewing.

Our results could not be explained by factors related to anatomical alignment nor by BOLD SNR fluctuations. First, we found no relationship between ISPC and anatomical misalignment as measured by the variance of convexity estimates at each vertex of reconstructed cortical surfaces. If the variance of ISPC were solely related to misalignment, we would have expected a strong negative correlation—greater anatomical misalignment should have correlated negatively with ISPC, but we did not observe such a trend. Our large sample size (54) strengthens this point, as our variability estimate would have been a robust estimate of population variance. The absence of a relationship between anatomical alignment and ISPC in this study corroborates an earlier study of the auditory cortex (Tahmasebi et al. 2012), where functional pattern variations in higher cognitive levels of the auditory cortex could not be explained by greater anatomical variation.

Because of the naturalistic stimuli used, it is possible that BOLD amplitude changes—due to the presence or absence of scene features important for a given cortical area—would induce the spatial pattern correlations we report here. Yet we found no support for this. We found that BOLD amplitude was a poor predictor of ISPC, as we were unable to find a single node cluster that survived FDR correction for multiple comparisons when testing the time-series correlation of BOLD amplitude and ISPC.

We determined that spatial pattern similarity is stimulus-driven and this was supported by 2 separate analyses. First, we tested whether spatial patterns induced by 1 movie explained patterns induced by another movie clip. A correlation between patterns induced by 2 movies would preclude stimulus-selectivity, but we failed to observe such a correlation. This first analysis thus supports the view that spatial pattern correlations are stimulus-driven.

Second, we further tested the degree of stimulus-selectivity by comparing spatial patterns and BOLD time series between 2D and 3D viewings of the same clip. As a stronger test of stimulus-selectivity, we would expect ISPC to be modulated by 3D versus 2D viewing. As a comparison, we took the BOLD time-series correlations between the same clips. We found that the BOLD time series was strongly correlated between the 3D and 2D viewing of the same clip, whereas the spatial pattern was substantially less correlated—spatial patterns were more sensitive to viewing condition than BOLD amplitude.

### Implications of ISPC Nonuniformity on Other fMRI Techniques

Our results lend support to alternative methods of functional brain mapping in which cortical areas are not treated as uniformly active in response to their preferred stimuli, such as the information-based method by Kriegeskorte et al. (2008). Traditional fMRI experiments heavily smooth spatial data to magnify areas of high amplitude signal due to the belief that the limited spatial resolution of fMRI could only distinguish which brain regions corresponded to which functions. Modern MVPA techniques pioneered by Haxby et al. (2001) and further supported by the present results demonstrate that smoothing the data is actually removing a major source of information about the brain’s function. The meso-scale spatial patterns presented here and traditionally removed by spatial smoothing, encode important information because they are shared among a large cohort of individuals during naturalistic stimulation. Most importantly, we demonstrate that significant ISPC can be found with anatomical alignment alone, without necessitating abstraction of the cortical response using RSA or functional alignment and at a much smaller spatial scale than previously noted.

One example of functional alignment, termed “hyperalignment,” involves the alignment of the spatio-temporal pattern in searchlights across the cortex (Sabuncu et al. 2010; Guntupalli et al. 2016). If some regions exhibit greater dissimilarity across subjects than other regions, then functional responses in these regions will need greater transformation. Because the purpose of the transformation is to facilitate multivoxel classification, our results suggest that these latter analyses may have an inherent and systemic bias depending on the region of the brain they are being carried out on. Due to greater ISPC values in V1, for example, responses in this area will need less transformation and hence less interpolation and blurring, yielding greater capacity for maintaining discriminant patterns. Beyond V1, most areas showed decreasing ISPC and would hence need greater functional alignment than lower areas. Another implication of our results on hyperalignment is the effect of searchlight size. Since whole-brain hyperalignment involves using searchlights of a consistent size across the brain and we demonstrate that searchlight size has an effect on ISPC scope (Supplementary Fig. 4), a variable searchlight method may be more accurate in preserving existing shared spatial structures of the cortex.

Our results align with existing bsMVPC studies, which show poor between-subject classification accuracies compared with within-subject, and even that poor performance being limited to a very small subset of early visual cortex (Guntupalli et al. 2016). This limited localization of bsMVPC performance relates heavily to areas with the highest magnitude of ISPC in our study, indicating that significant accuracies can be achieved with sufficient intersubject similarity in the spatial pattern of response. Our results go on to show that significant pattern similarity can also be found in much of the visual cortex, but to differing degrees. Future studies may incorporate nonuniform similarity and searchlight size requirements from our study to create more accurate bsMVPC experiments without the need for further transformations to the response.

### What Causes Variations in ISPC?

Most of the visual cortex exhibited spatial pattern similarity except IPS1–IPS4, SPL1, and PHC1 and 2. SPL1 has been found to be involved in saccades (e.g., Konen and Kastner 2008), and we speculate that the very low correlation values in this region were due to our subjects fixating. IPS1–IPS4 are also involved in the representation of eye movements and the lack of spatial pattern correlation in these areas could be similarly explained (Arcaro et al. 2009). The remaining visual cortex exhibited nonuniform spatial pattern similarity.

We could conceive of 3 hypotheses to explain variations in spatial pattern similarity, which we termed: Experiential Cortex, Receptive Field, and Motif Size hypotheses. According to the Experiential Cortex hypothesis, shared spatial patterns may reflect regions that are not shaped by individual’s unique experiences—areas with high spatial pattern correlation reflect areas whose structure is largely predetermined by genetic factors. In contrast, the Receptive Field hypothesis predicts spatial pattern similarity is related to the population receptive field size of a given area—larger receptive field sizes (and correspondingly weaker retinotopic organization) result in smoother variations across the cortical surface, reflected in lower ISPC values across individuals. Finally, according to the Motif Size hypothesis, the variation we observed in spatial structural similarity is reflective of the “size” of the underlying spatial motifs—for higher level areas, these motifs may be larger than our searchlights, and hence the estimated spatial pattern similarity is not uniform across the cortex when sampled by a single searchlight size.

We did not find evidence supporting the Experiential Cortex hypothesis, which would predict that high spatial pattern similarity coincides with areas that are not strongly shaped by individual experience. V1 and V2 are areas with this property, having a predictable functional structure, which can be estimated from anatomy (Burkhalter 1993; Benson et al. 2012; Benson et al. 2014). Our results depict high ISPC in V1 and V2 compared with other visual areas; however, there was a large difference in magnitude between them (over 50%), suggesting other factors must be involved. The Receptive Field hypothesis is weakly supported by our results. The estimated map of population receptive field sizes presented by Dumoulin and Wandell (2008) does not appear to correspond to our ISPC map, though there are some similar trends. For example, the population receptive field of lateral occipital cortex (LO)/ventral occipital cortex (VO) was found to be roughly 5 times larger than V1—a similar trend was found in our study for spatial pattern similarity.

Lastly, we found strong evidence to support the Motif Size hypothesis. Results from 3 searchlight sizes revealed that only when sampling with larger searchlights do higher-level visual areas become significantly similar (see Supplementary Fig. 4), suggesting higher visual areas contain larger shared patterns than early areas. This finding is in accordance with results from Haxby et al. (2001), which suggest that ventral temporal cortex represents objects with distributed, overlapping patterns, and hypotheses from Op de Beeck et al. (2008), stating that the nonlinear combination of multiple feature maps may give rise to a distributed pattern responsible for diverse object recognition. It is also possible that some of the stronger ISPC found with larger searchlights is a consequence of including more than 1 selective object recognition modules in 1 searchlight, such as the fusiform face area and parahippocampal place area. We do not believe this to be a strong driving factor because our stimulus does not include many of the objects typically described to excite these areas selectively (I.e., human body parts, human faces, buildings; except possible faces of sea animals), our searchlights are relatively small, and our effect was not limited to ventrolateral occipital cortex. Along with our results on the stimulus-driven nature of the spatial patterns described, this suggests that higher visual areas recruit more distributed spatial structures responsible for the processing of visual information. In contrast, V1 is made up of a repeating pattern of feature detectors—small spatial structures—that cover the entire visual field retinotopically (e.g., orientation columns repeat the same spatial motif, called pinwheels, over entire V1). A small searchlight will capture similar patterns across subjects in V1 because the small spatial structures will be activated the same way across the retinotopic map across all subjects. When a larger searchlight is used, more of these repeated small structures will be captured, so spatial pattern similarity will be conserved.

Current hyperalignment and whole-brain MVPA techniques use consistently sized searchlights across the cortex to examine local spatial patterns and make inferences about the population. We have shown that different searchlight sizes affect ISPC and hypothesize that different searchlights may be capturing different spatial motifs. Future studies could test different searchlight sizes concurrently across the cortex, or sequentially testing a few, before determining the best searchlight method for their study.

Because we found ISPC to be stimulus-driven, it is also nonuniform across time. We speculate that the dynamic aspect of ISPC is related to saliency (Niebur et al. 1998). Saliency limits the processing demands of the visual system to smaller parts of the visual field for efficiency and speed and is driven by prominent features of the stimulus, such as high contrast or vivid color (Koch and Ullman 1985). Although our subjects were fixating, they were still able to covertly shift their attention to salient regions of the scene. In the case of V1, a shift in saliency will also shift the main feature-detecting pattern on V1 to a different retinotopically corresponding portion of V1 (Brefczynski and DeYoe 1999). In the case of higher-level ventral areas, a shift in saliency will not change location of the pattern, due to weak retinotopic mapping in these areas, but instead will fundamentally change the pattern itself because different maps will be activated to form the unique combined patterns representing the object/scene (Treue and Trujillo 1999; Downing et al. 2001).

### ISPC Compared to ISC

Our ISPC values are lower and cover less of the cortex than previous studies of temporal similarity using the ISC method (Hasson et al. 2004; Chen et al. 2016b). Cinematic movies contain many nonvisual temporal structures, such as suspense and executive load driven by the plot of the movie. These aspects of the movie have been shown to drive synchronous activity in the frontal and parietal lobe (Naci et al. 2014). We used a vivid, non-narrative movie and restrained our investigations to the visual system only, which likely removed a significant amount of synchronicity across the cortex found in previous studies. Although potentially limiting, this strategy is a crucial first step to examining common spatial structures of the visual system because top-down influences may also drive similarity unrelated to underlying structure.

Temporal ISC and ISPC are at least partly independent of one another. When BOLD amplitude is relatively weak in a region, the temporal pattern will not be robust enough to find similarity but the spatial pattern can still be significantly similar across subjects (Haxby et al. 2001; Sterzer et al. 2008). Future studies will need to incorporate both temporal and spatial ISC during naturalistic stimulation to better capture the representations underlying the fMRI response.

## Conclusion

The visual system encodes everyday experience in similar spatial patterns across individuals. Shared spatial patterns vary by location in the visual cortex, the size of the spatial patterns sampled, and the movie content and complexity (3D vs. 2D). They carry different information and represent stimulus-driven activation across the visual cortex even more so than voxel-wise temporal BOLD signal alone. Future studies may benefit from our improved understanding of intersubject spatial pattern similarity. Nonuniform similarity across the cortex implies that inherent biases may be present when treating them as uniform, from the rudimentary step of aligning and smoothing different brains, to functional alignment, to studying complex brain functions using MVPA.

Spatial patterns may also serve as an indicator of normal cortical behavior, similar to how temporal patterns have been investigated in individuals with depression, schizophrenia, and autism (Hasson et al. 2009; Guo et al. 2015; Gruskin et al. 2020; Tu et al. 2019). Combining these techniques to incorporate the full spatio-temporal response pattern will allow future researchers to localize subtle functional changes in the brain while sampling many visual features at once, in a naturalistic visual environment most like everyday experience, making it extremely practical and impactful for patients’ lives. In sum, a map of spatial pattern similarity values across the cortex provides the basis for predicting prototypicality of spatially patterned brain responses in any individual and contributes to building a general blueprint for human brain function.

## Notes

We thank Sebastien Proulx and Yiran Chen for their valuable input during the design and execution of our analysis techniques. *Conflict of interest:* The authors declare no competing interests.

## Funding

FRQS Vision Health Research Network Common Infrastructure Program; Canadian Institutes of Health Research (CIHR) (grant 378590 to R.F.)

## Supplementary Material

Final_Supplementary_tgaa076
