# Supplementary material for: Intersubject Spatial Pattern Correlations During Movie Viewing Are Stimulus-Driven and Nonuniform Across the Cortex
Source: Cereb Cortex Commun. 2020 Oct 23;1(1):tgaa076. doi: 10.1093/texcom/tgaa076 (PMC7679429; doi:10.1093/texcom/tgaa076)
Supplement: Final_Supplementary_tgaa076 [file final_supplementary_tgaa076.docx]

#
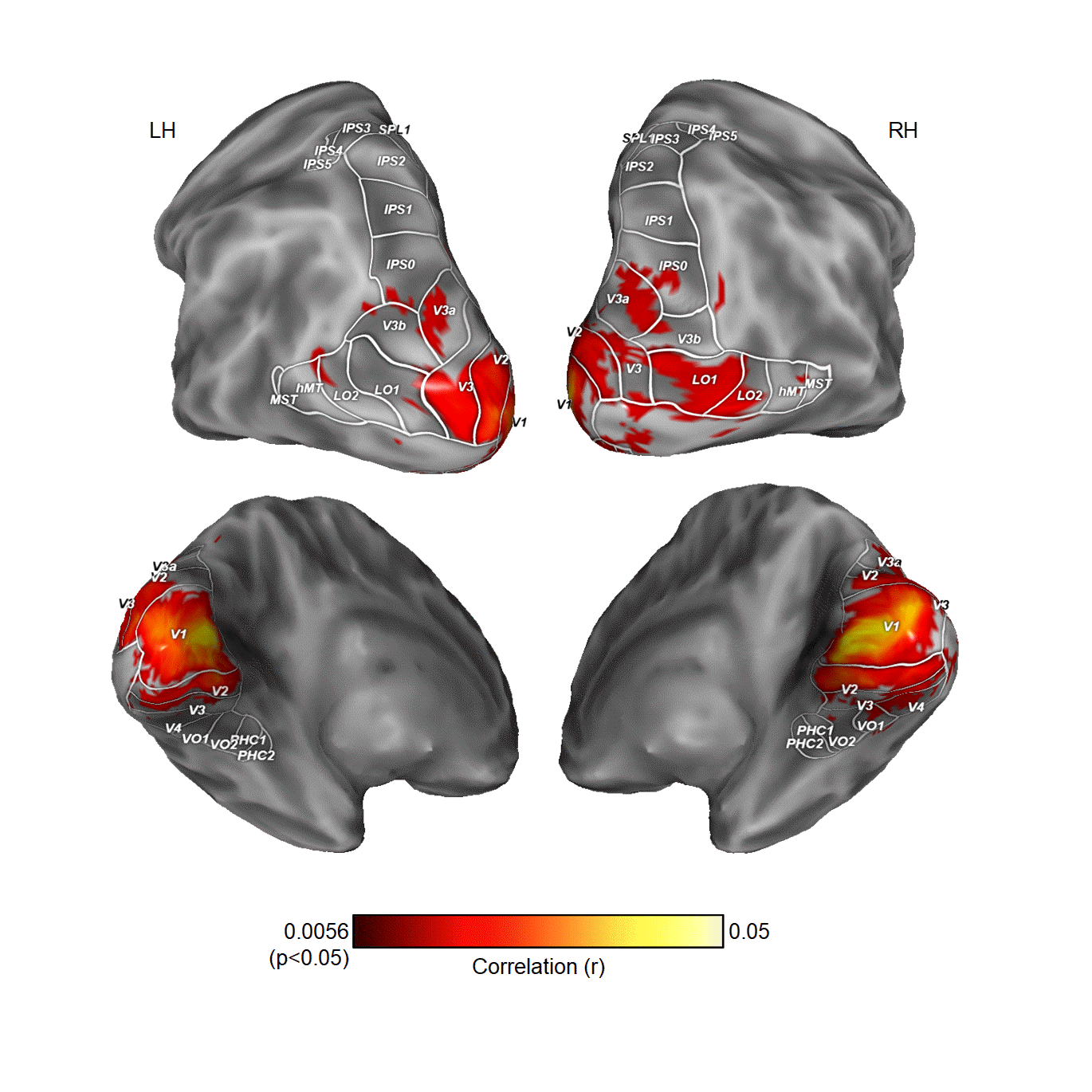


**Supplementary Figure 1.** Spatial pattern similarity across subjects in 2-D version of clip 1, analyzed with 5mm searchlights (N=54). Significant similarity remains non-uniform but covers less of the visual cortex than in larger searchlight sizes and limited primarily to early visual areas and right lateral occipital areas (LO1 and LO2). Only areas that passed the family-wise error corrected threshold from permutation tests are shown. Visual area borders reflect population atlas boundaries^33^.


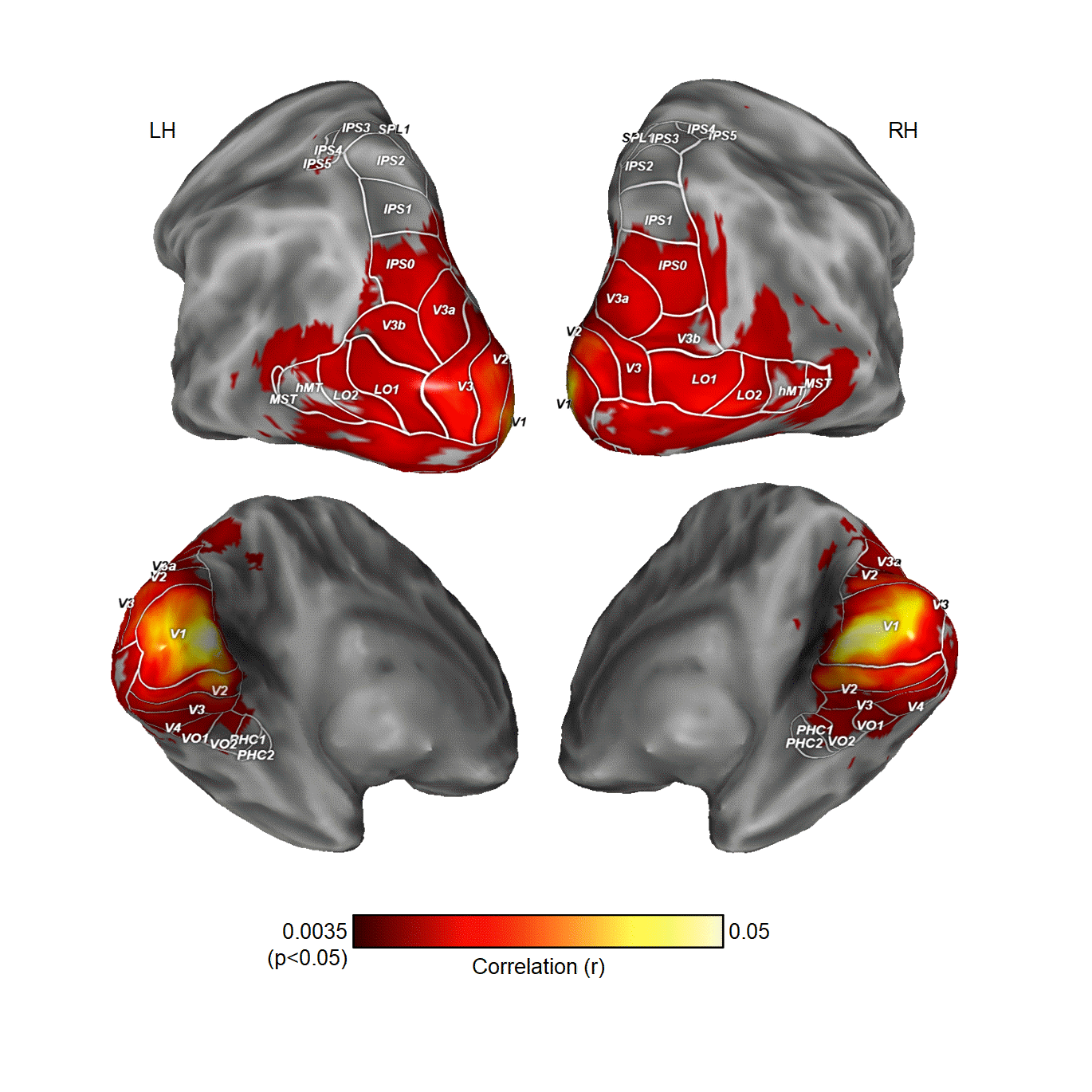


**Supplementary Figure 2.** Spatial pattern similarity across subjects in 2-D version of clip 1, analyzed with 7mm searchlights (N=54). Significant similarity remains non-uniform and covers more of the visual cortex than with 5mm searchlights but less than with 9mm searchlights. Only areas that passed the family-wise error corrected threshold from permutation tests are shown. Visual area borders reflect population atlas boundaries^33^.


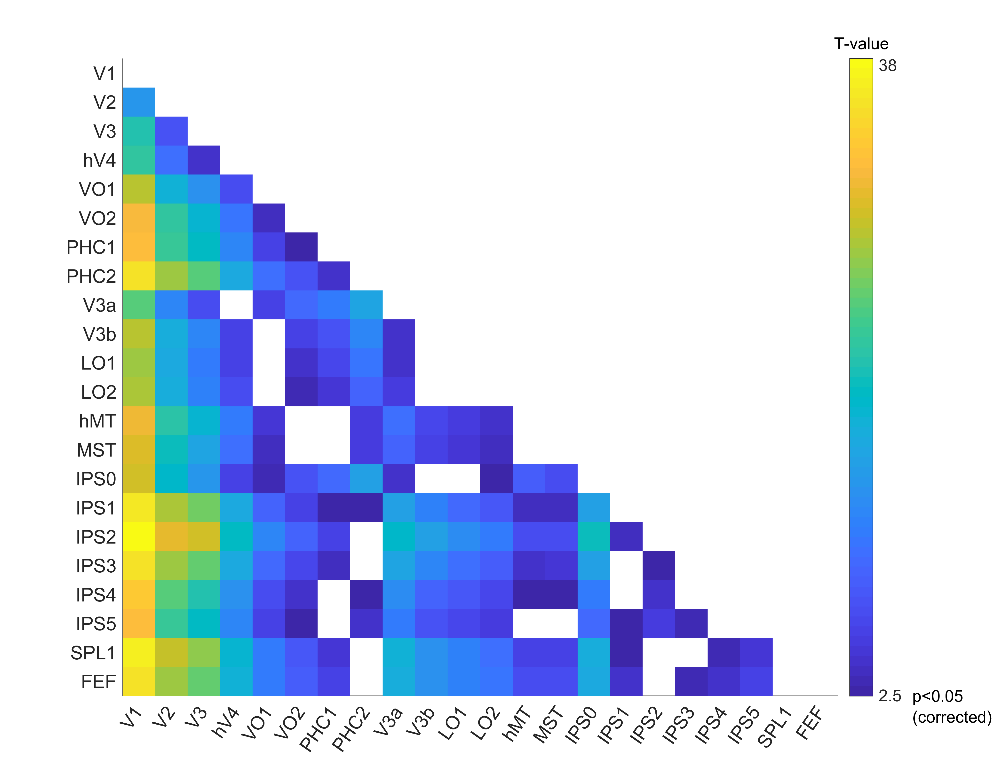


**Supplementary Figure 3.** Heatmap of T-values from two-sample t-test of subsampled groups for comparisons between visual areas using N=27 sub-samples of 53 pairs each, thresholded at *p*=0.05 (corrected).

**
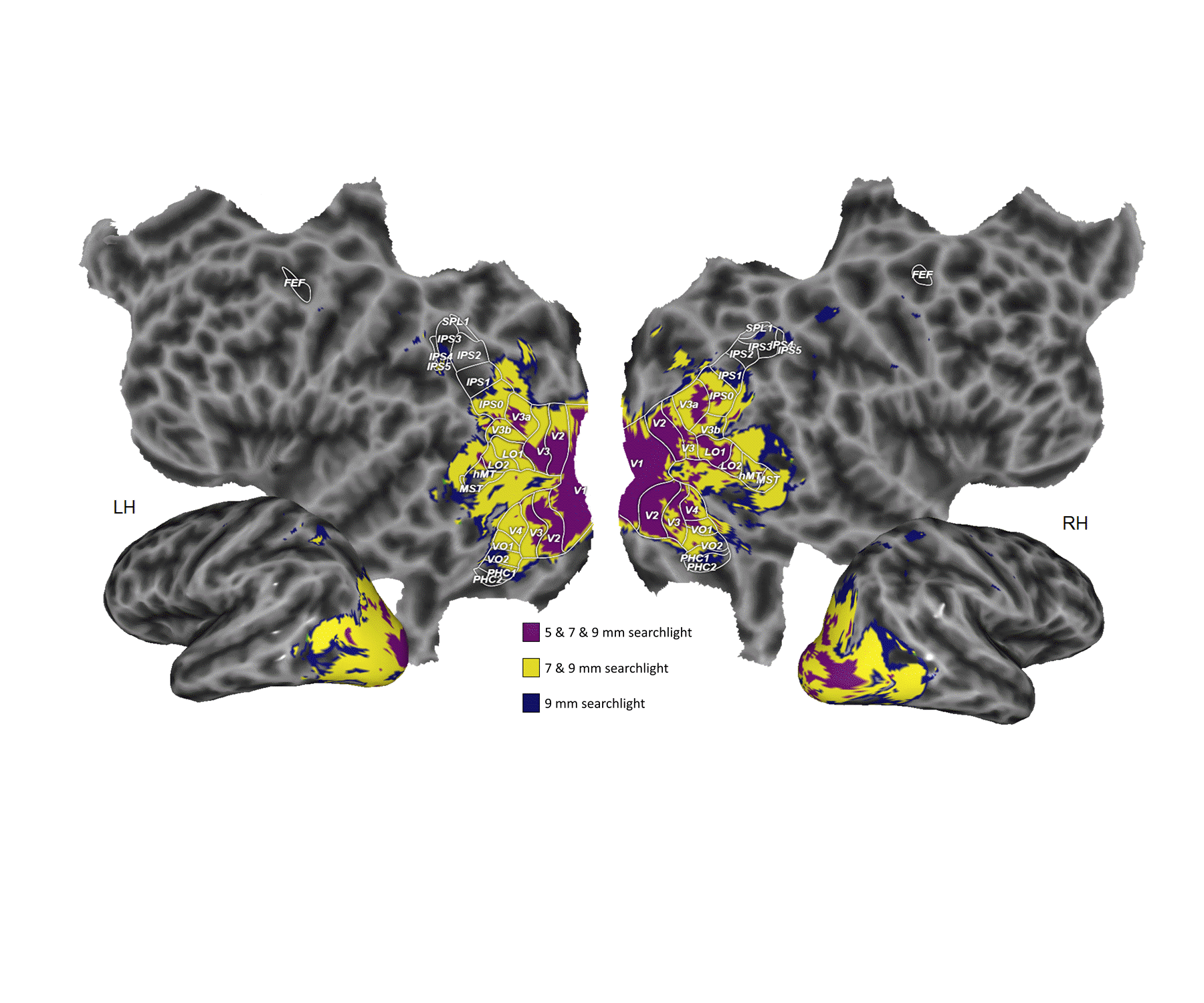
Supplementary Figure 4.** Area of cortex with significant spatial pattern similarity for each searchlight size (5mm, 7mm, 9mm) from 2-D version of clip 1 (N=54). Larger searchlights cover all the area of smaller searchlights, while additionally spreading to higher order areas.
